# A Bayesian Approach to Quantifying the Effects of Mass Poultry Vaccination upon the Spatial and Temporal Dynamics of H5N1 in Northern Vietnam

**DOI:** 10.1371/journal.pcbi.1000683

**Published:** 2010-02-19

**Authors:** Patrick G. T. Walker, Simon Cauchemez, Raphaëlle Métras, Do Huu Dung, Dirk Pfeiffer, Azra C. Ghani

**Affiliations:** 1 MRC Centre for Outbreak Analysis Modelling, Department of Infectious Disease Epidemiology, Imperial College London, London, United Kingdom; 2 Veterinary Epidemiology and Public Health Group, Department of Veterinary Clinical Sciences, The Royal Veterinary College, North Mymms, United Kingdom; 3 Department of Animal Health, Hanoi, Vietnam; University of Texas at Austin, United States of America

## Abstract

Outbreaks of H5N1 in poultry in Vietnam continue to threaten the livelihoods of those reliant on poultry production whilst simultaneously posing a severe public health risk given the high mortality associated with human infection. Authorities have invested significant resources in order to control these outbreaks. Of particular interest is the decision, following a second wave of outbreaks, to move from a “stamping out” approach to the implementation of a nationwide mass vaccination campaign. Outbreaks which occurred around this shift in policy provide a unique opportunity to evaluate the relative effectiveness of these approaches and to help other countries make informed judgements when developing control strategies. Here we use Bayesian Markov Chain Monte Carlo (MCMC) data augmentation techniques to derive the first quantitative estimates of the impact of the vaccination campaign on the spread of outbreaks of H5N1 in northern Vietnam. We find a substantial decrease in the transmissibility of infection between communes following vaccination. This was coupled with a significant increase in the time from infection to detection of the outbreak. Using a cladistic approach we estimated that, according to the posterior mean effect of pruning the reconstructed epidemic tree, two thirds of the outbreaks in 2007 could be attributed to this decrease in the rate of reporting. The net impact of these two effects was a less intense but longer-lasting wave and, whilst not sufficient to prevent the sustained spread of outbreaks, an overall reduction in the likelihood of the transmission of infection between communes. These findings highlight the need for more effectively targeted surveillance in order to help ensure that the effective coverage achieved by mass vaccination is converted into a reduction in the likelihood of outbreaks occurring which is sufficient to control the spread of H5N1 in Vietnam.

## Author Summary

Highly pathogenic avian influenza H5N1 continues to spread rapidly between flocks of poultry in many parts of the world including areas in Southeast Asia and Africa where infection has become endemic. Meanwhile the number of human cases and fatalities are steadily accumulating. As a result, the control of outbreaks in poultry remains both a key public and animal health priority. In Vietnam control policies have evolved from a policy of reliance upon drastic “stamping out” measures to regular mass vaccination campaigns. Using Bayesian data augmentation techniques in order to take into account the unobserved infection times, we found that this has led to a significant reduction in the daily probability of transmission between communes but that the time taken to detect outbreaks has increased. As a result it is still possible for sustained transmission to occur, albeit at a slower rate. Through an analysis of the reconstructed epidemic tree, we found that any measures that have the effect of restoring detection capacity to that estimated for the “stamping out” policy would have a large effect upon the size and scale of any future outbreak wave and may be effective at preventing sustained transmission between communes.

## Introduction

Highly pathogenic avian influenza subtype H5N1 was first identified in Vietnam in December 2003 [1] and was followed by a major wave of outbreaks dispersed widely throughout the country around the 2004 festival of Tê′t, a New Year celebration representing a peak in annual poultry production and consumption. Measures employed to control both this and a second wave of outbreaks, occurring a year later, included a ‘stamping out’ policy with compulsory mass ring culling of all poultry around an outbreak carried out. This led to over 40 million poultry being culled during the first wave alone, contributing to an estimated direct national loss of US$ 200 million [2], a sharp decline in demand for poultry products [3] and an estimated loss to affected individual stakeholders of between US$ 69 and US$108 [4], an amount exceeding the average monthly wage in Vietnam.

The resources and scale of destruction necessary to maintain these measures and the repeated incursions of H5N1 which occurred despite them resulted in a shift in policy in 2005 [5]. Following a field trial and pilot campaign in two test provinces, a nationwide mass vaccination campaign began, using an H5N2 vaccine to inoculate chickens and a recombinant H5N1 vaccine for ducks [6], with priority given to recently affected areas and those with high human and poultry densities [7]. Approximately 160 million doses were administered twice a year costing close to $US 21 million [8]. Although a third wave of outbreaks coincided with the beginning of this campaign, a sero-survey of poultry following vaccination estimated that a 60% level of protective coverage had been achieved [6]. A nationwide ban on the hatching of waterfowl was also enforced from February 2005 [7].

Following this third wave, Vietnam experienced a year during which no outbreaks were reported. Despite follow-up vaccination campaigns from August to November 2006 and March to June 2007, a fourth wave of outbreaks occurred in the South of Vietnam in December 2006 and a fifth, beginning in late April 2007, in the North, coinciding with the lifting of the ban on duck hatching. During both of the latter waves, in order to minimise losses to farmers, ring vaccination was carried out around each reported outbreak and only flocks within which infection had been identified were culled [2]. Detailed records were not kept during the first two weeks of the first wave [5]. However, following the implementation of systematic community-based and veterinary surveillance and reporting networks as part of a detailed National Action Plan [9], temporal and spatial outbreak data were collected at a commune-level resolution for all subsequent waves, providing a unique opportunity to assess the effects of the interventions, and in particular country-wide vaccination.

**Figure 1:**
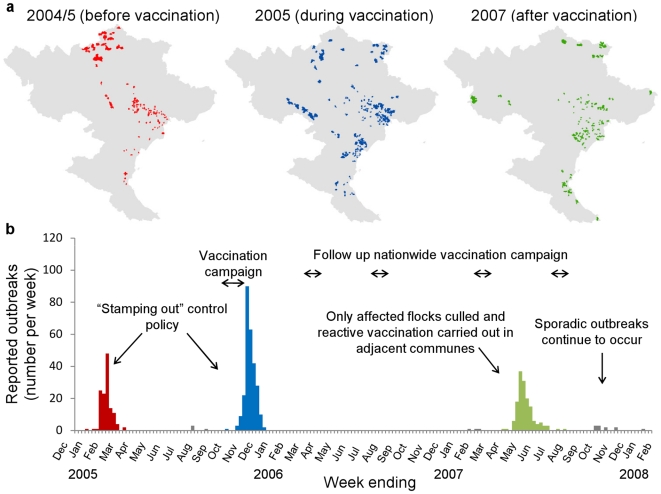
Spatial and temporal distribution of outbreaks of H5N1 in poultry in northern Vietnam Dec 2004–Feb 2008. a, Commune-level spatial distribution of reported outbreaks, b, Weekly incidence of outbreak reports in northern Vietnam. (As sufficiently accurate data on the timing and location of outbreaks during the first wave is not available, figure shows data from the second wave onwards).

Here we focus upon northern Vietnam, where data were available for the sizeable waves occurring before, during and after vaccination was implemented, with outbreaks generally concentrated around the high risk Red River Delta (RRD) region (Fig. 1). However the analysis of this data is challenging because of the inherent dependency between infection events (i.e. the risk of infection of one commune depends on the infection status of all neighbouring communes) and missing data issues (the time of first infection of a commune is not known as only reporting dates have been recorded). We therefore developed an inter-commune transmission model, characterised by a spatial kernel describing how infectivity scales by distance and a parametric infection-to-report distribution, in order to capture the spatial and temporal dynamics of the spread of infection in the context of Vietnam where the smallholding of poultry is commonplace. Bayesian MCMC data augmentation methods which treat infection times as nuisance parameters [10]–[14] were then used in order to impute the missing infection times and fit this model to the observed commune-level outbreak reports. This allowed us to explore the changes which occurred between waves, both in terms of the transmissibility between communes and the rate at which outbreaks were detected. Next, through an analysis of the possible chains of transmission (the “epidemic tree”), we investigated how changes in the rate of reporting outbreaks affected the size and duration of the wave of outbreaks which occurred following the implementation of vaccination.

## Results

**Figure 2:**
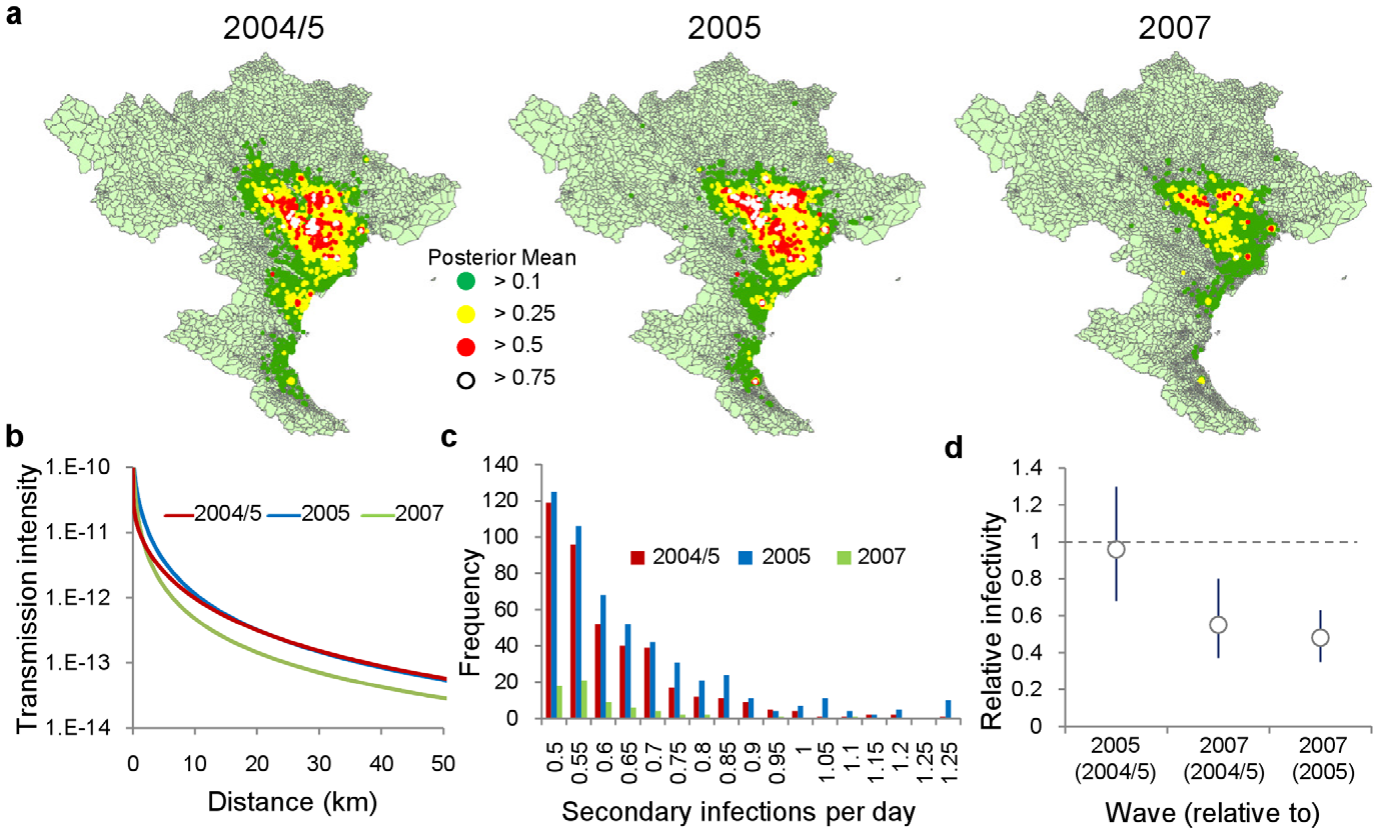
Between-commune infectivity. a, Risk map of the expected daily number of secondary infections arising from infected commune during each of the three waves, under the assumption all other communes remain susceptible. b, Plot of how the daily probability of between-commune transmission scales with distance, c, Histogram of commune-specific daily rate of infection (only those over 0.5 plotted), d, Relative changes in per-capita infectivity parameter 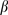 between waves with associated 95% credible intervals (see *Text S1*).

Our results suggest that the expected daily number of secondary outbreaks generated by one infected commune varied little between the waves occurring before and during the vaccination campaign. However, during the 2007 wave, following vaccination, this measure of spread reduced substantially as a result of a significant reduction in the daily probability of transmission between communes (Fig. 2a–c), with a joint posterior mean estimate suggesting that, per head of poultry, infectivity was only 55% of that during the 2004/5 wave (Fig. 2d). Making the assumption that changes in infectivity are caused by vaccination and not influenced by other factors including changes in the effectiveness of other control measures such as bio-security or movement restriction, this can be compared to the effects of a vaccination campaign achieving 55% uniform effective coverage (where effective coverage refers to the overall vaccination coverage multiplied by the protective efficacy of the vaccine).

**Figure 3:**
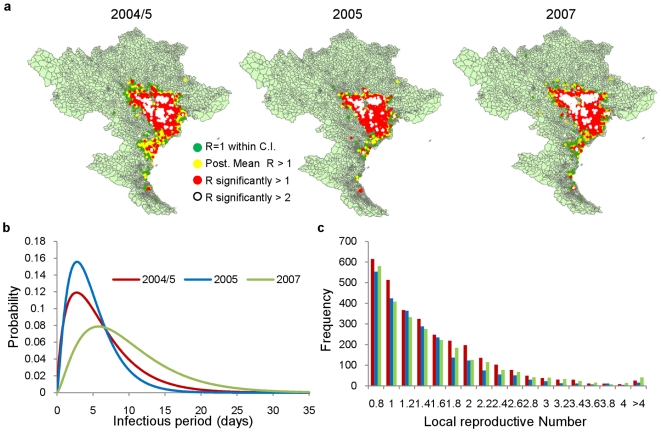
The infectious period and local reproductive numbers. a, Spatial distribution of local reproductive numbers calculated analytically using the infectivity and infectious period estimates, b, Estimate of probability (assumed gamma) distribution of the infectious period during each of the three waves, c, Histogram of local reproductive numbers for the three waves (only those with values greater than 0.8 plotted).

**Figure 4:**
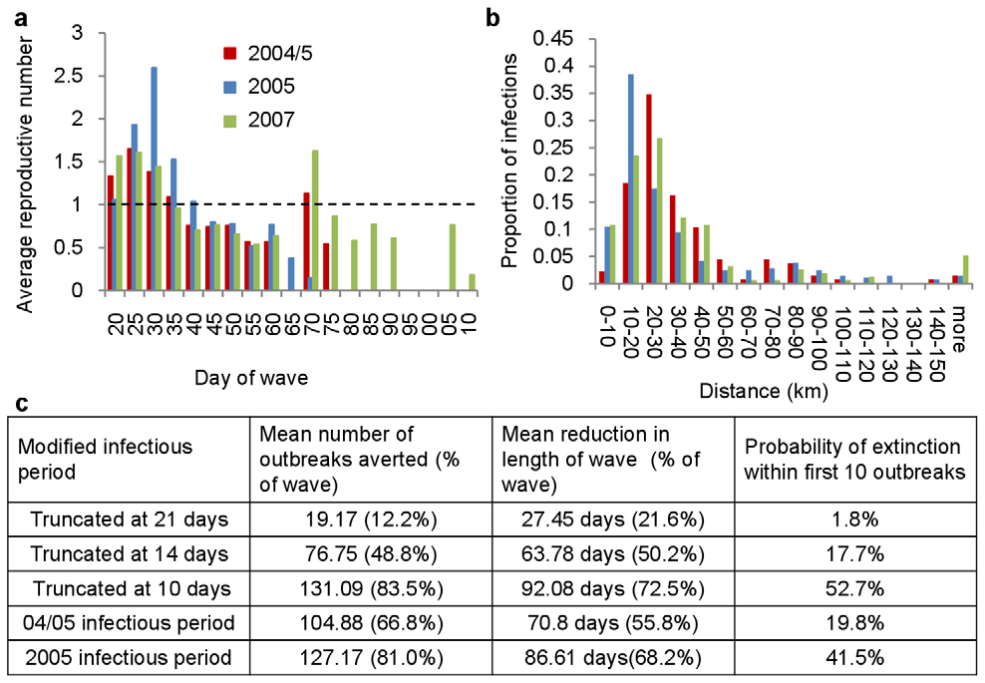
Reconstructing the epidemic tree. a, Temporal pattern of average reproductive numbers throughout the three waves, b, Proportion of transmissions occurring at different distances for each of the three waves, c, Impact upon the 2007 wave of truncating the infectious period after a set amount of time if an outbreak remains unreported, reflecting the time at which infection would be detected using more pro-active surveillance. Columns show the estimated number of outbreaks which would have been averted as a result, the reduction in the time taken to control the wave and the probability the wave would have been eliminated within the first ten outbreaks.

In contrast, the infectious period of communes increased significantly during the 2007 wave (Table 1 and Fig. 3b). Consequently, despite the coincident reduction in the daily transmission probability, the RRD region continued to sustain infection (Fig. 3a and c), resulting in a wave which was lower in intensity but longer lasting than the preceding two waves (Fig. 4a). Overall, accounting for changes in the distribution of poultry between the 2004/5 and 2007 waves, there was an 11% reduction in the number of communes with estimated local reproductive numbers (see *Methods* *and Text S1*) above unity The posterior mean commune-level infectious period decreased from an estimated 5.9 days during the 2004/5 wave to just 4.7 days during the 2005 wave (Fig. 3b), coinciding with an increase in the level of compensation provided to farmers with infected or depopulated flocks [15]. As a result, despite the fact infection spread more rapidly in the initial stages, the wave was brought under control more rapidly than during the other two waves (Fig. 4a). There was also a high proportion, relative to the other waves, of transmission taking place over distances of less than 20km (Fig. 4b).

**Table 1:** Posterior mean and 95% credible intervals for the baseline model fitted to each of the three outbreak waves.

Model Parameters		2004/5	2005	2007
Parameter	Description	Posterior mean (95% C.I.)		
**Spatial Transmissibility**				
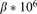	Intensity	3.96 (0.45–14.5)	1.85 (0.75–4.88)	2.06 (0.57–6.02)
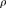	Offset	3.46 (0.36–13.68)	0.83 (0.22–3.42)	0.77 (0.04–2.21)
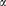	Power	2.14 (1.57–3.07)	2.02 (1.75–2.35)	1.81 (1.60–2.08)
**Duration between infection and report**				
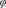	Shape	1.81 (1.24–2.75)	2.33 (1.71–3.21)	2.45 (1.49–4.54)
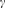	Scale	3.37 (2.04–5.01)	2.09 (1.33–3.12)	3.98 (2.11–6.15)
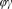	Mean	5.87 (4.59–7.40)	4.74 (3.84–5.91)	9.10 (7.29–11.21)
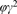	Variance	20.04 (10.25–34.82)	10.09 (5.29–17.80)	36.29 (18.69–60.14)

We assessed the robustness of our qualitative conclusions about the changes in the dynamics of infection following vaccination to different assumptions about the infectious period and effects of control measures with detailed sensitivity analyses (see *Text S1*). We were unable to explain the reduction in infectivity and increase in the infectious period following vaccination by allowing outbreaks to remain infectious for a longer time following a report or by modelling non-constant infectivity throughout the infectious period. We also found that the estimated reduction in the daily probability of transmission (Fig. 2b) could not be attributed to a decrease in the proportion of outbreaks detected.

Treating each outbreak as the root of a separate sub-epidemic or “clade” [16], where a clade is defined to consist of all future generations of outbreaks arising from an individual infection (see *Methods* and *Text S1*), we explored the impact more effective surveillance would have had upon the 2007 wave of outbreaks. We made the assumption that this would have primarily resulted in the earlier reporting of outbreaks estimated to have remained unreported for the longest time.. We found that, had all outbreaks been identified and successfully removed within two weeks of the initial infection, the expected eventual size of the wave would have been halved and the wave would have been eliminated twice as fast, with an 18% probability that the wave would have become extinct within the first 10 outbreaks. This probability rises to 20%, with a 67% reduction in the size of the wave, if the rate of detection had been maintained at that estimated for the 2004/5 wave (Fig. 4c), prior to the implementation of vaccination.

## Discussion

Our results demonstrate the application of data augmentation techniques to existing livestock disease modelling methodologies in order to quantify the spatial and temporal spread of disease in a setting where the times of infection are unobserved. We found that, following the implementation of vaccination, the day-to-day probability of infection spreading between communes has been significantly reduced, with our estimate of a 55% effective vaccination coverage agreeing with post-campaign sero-surveillance [6]. However, we also found that the duration of time taken to report outbreaks had also increased significantly, allowing infection to spread. This result may support the hypothesis that vaccination within a flock can contribute to the “silent spread” of infection whereby a low-level of flock mortality or asymptomatic infections can make outbreaks more difficult to detect [17],[18] and this was identified locally as an exacerbating factor in the spread of infection [19].

There does, however, also appear to have been a shift in the distribution of host species involved in outbreaks. During the 2004/5 wave, only 30% of outbreaks were identified in ducks whereas in 2007 this figure rose to 77% [7]. This change has previously been attributed to the lifting of a duck-hatching ban in February 2007. Scavenging ducks traditionally play an important role in the poultry production system and they have previously been identified as being associated with outbreaks of HPAI [7]. Outbreaks also coincided with the end of rice-harvest season when ducks are allowed to graze freely on rice paddies [20]. H5N1 infection in ducks has been shown to be less pathogenic than in chickens, with clinical signs being less apparent and slower to develop [21],[22]. As a result, the concentration of infection within duck flocks may have contributed to an elongated commune-level infectious period. Changes in the transmissibility or reporting rates of outbreaks may also be linked with other factors such as differential vaccine efficacy or observed changes in the genotype of the virus [23].

We found that a large proportion of transmission occurring concurrently with the first round of vaccination took place over relatively short distances. Such an increase could be due to the movement of vaccinators who, it has been suggested, may have acted as carriers of infection from one commune to another [5]. It is also interesting that our estimates didn't suggest a reduction in the daily per-capita poultry transmissibility 2005, despite the concurrent vaccination campaign which was taking place. This may suggest that outbreaks predominantly occurred within large regions yet to be vaccinated or in areas where vaccinated poultry had yet to develop effective immunity.

We demonstrated how imputed infection times can be used to explore all possible chains of infection and to assess the range of possible effects which can be achieved by ‘pruning’ an outbreak from the epidemic tree. Applying this to outbreaks occurring during the 2007 wave, we found that if the reporting rates had been maintained to those achieved during the previous waves, the size and the duration of wave would have been considerably diminished. This highlights that, in order to ensure that these reductions in infectivity achieved through vaccination (Fig. 2a,c,d) are translated into a noticeable impact on outbreak size, it is essential that the infectious period of communes is shortened (Fig. 3b) via improved surveillance and reporting. Measures towards this have already been attempted in Vietnam with the trial of a pro-active surveillance programme whereby resources are allocated according to known risk factors such as the presence of live bird markets and high semi-commercial farm density and a prior history of outbreaks [24]. The programme also includes an awareness campaign informing stakeholders about how outbreak detection criteria should change following the vaccination of a flock. An alternative approach is to use unvaccinated birds, or those vaccinated with an inactivated heterologous strain of avian influenza, as sentinels for the detection of the spread of infection. This has been implemented under the Differentiating Infected from Vaccinated Animals (DIVA) guidelines in developed countries such as Italy and the U.S. and has been successful in helping to control outbreaks of low pathogenic avian influenza (LPAI) [25],[26].

Given the size of, and low-levels of biosecurity within, the poultry industry and the ubiquity of poultry-keeping in Vietnam [27], developing such detection capacity will involve overcoming a large variety of logistical, economic and social issues [3]. However, in the absence of such measures, sporadic outbreaks of the kind currently being experienced in Vietnam, and the public health risks they entail [28], seem set to re-occur for the foreseeable future.

## Methods

Outbreaks of H5N1 in Vietnam prior to 2008 generally occurred around two main foci, the Red River Delta in the North of the country and the Mekong River delta in the South. Factors such as the differing climates and poultry grazing patterns [5], as well as the distinct genotypic profile of outbreaks which occur within these regions [23] mean that transmission risk factors and intensity are likely to vary between each region. As a result we restrict our analysis to the Northern Red River Delta foci, the region which has experienced the largest number of waves during our study period. We define this region as the area of Vietnam which lies north of the 18^th^ latitude line, an area which comprises 6278 communes. We then defined three distinct waves of outbreaks, the first beginning in 2004 before the initiation of the vaccination campaign (2004/5 wave, 129 affected communes), a second beginning in October 2005, coinciding with the first round of vaccination (2005 wave, 271 affected communes) and a third occurring in 2007 more than a year after vaccination had become fully established (2007 wave, 152 communes affected).

We modelled these three waves as a spatio-temporal survival process [29], with communes remaining susceptible until the day of infection, becoming infectious from the day following infection and then removed from the process either through mass culling (for outbreaks occurring before the instigation of the vaccination campaign) or a combination of ring vaccination and the culling of infected flocks (following vaccination). Infectivity was assumed to remain constant throughout the infectious period, with transmission occurring according to a per-bird intensity 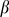, which scaled by distance from the source of infection according to a two-parameter spatial kernel,
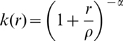
with the time between the infection of a commune and the report of an outbreak assumed to follow a gamma distribution. The scale and shape parameters of this distribution, the three spatial infectivity parameters, and each individual infection time were fitted to the observed outbreak reports for each wave using a two-step Metropolis-Hastings algorithm in order to obtain sufficiently large samples of the posterior distributions. These were then used to calculate the reproduction number of each commune and to reconstruct and investigate the infection tree in a probabilistic manner, allowing us to explore the possible effects of greater detection capacity.

### 

#### Data

Data on the outbreaks of H5N1 in Vietnam between March 2004 and February 2008 were collected by the sub-Departments of Animal Health for each of 64 provinces and city municipalities in Vietnam and combined into a single database by the Department of Animal Health in Hanoi. The database contains the smallest administrative unit (hereafter referred to as a commune although this can also be a town or ward) within which the outbreak occurred and the date upon which each outbreak was reported.

Spatial data in the form of a shapefile of the administrative boundaries of Vietnam, to a commune-level resolution, were obtained by the DAH from the Vietnamese Ministry of Natural Resources and Environment, as well as data on the human population of each commune according to 2001 census data, and annual poultry numbers at a provincial level resolution, obtained from the General Statistics Office of Vietnam website.

Some infected communes reported multiple outbreaks during a single wave. As the available data is at a commune level, we are forced to ignore the possible impact of within-commune epidemic dynamics. Therefore, based upon commune-level incubation periods obtained from previous waves, we made the assumption that in the case that a commune reported a secondary outbreak within 14 days of a previous report the later report was attributable to the earlier outbreak and the communes was assumed to have remained infectious throughout. However, in a few cases (5/129 communes during the 04/05 wave, 8/271 in 2005 and 4/152 in 2007) outbreaks were reported more than 14 days apart. These outbreaks were allowed to occur from external sources, the poultry population of the commune was then split in half and outbreak locations were generated randomly within the commune.

#### Parameter estimation

Each commune 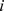 was assigned a location 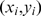, calculated as the centroid of the commune, a date of first infection 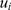 and a date of removal 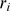 from the outbreak wave as a result of control measures or natural extinction (for ease of notation, any commune which is not infected during an outbreak wave is assigned an infection and removal time which is arbitrarily later than the final outbreak removal time 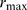). Vectors associated with these times 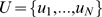 and 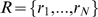
were also defined, with 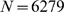 the number of communes in the dataset north of the 18^th^ latitude line. Communes were also assigned a poultry population 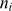, a fraction of the province-level poultry population proportional to the number of people living in the commune.

From this we defined the instantaneous force of infection experienced by a commune 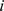 at time 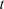:
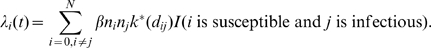
Here 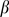 is a third infectivity parameter to be estimated and 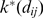 is the value of the appropriately normalised kernel at 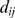, the straight-line distance between communes 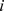 and 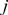. Thus the probability of avoiding infection until time 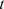 is 
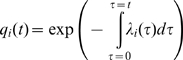
and the probability the commune is infected on day 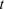 is 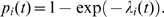
If the times of infection and removal were observed then the likelihood of the outbreak wave could be calculated as:
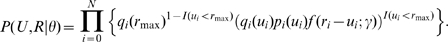
Here 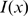
is the indicator function, 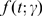
is an assumed gamma distribution of the duration of time between a commune first becoming infected and the date an outbreak is reported (the contribution of which to the likelihood, if infection and removal times were observed, would be a constant multiplier) and 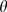 denotes the set of kernel and duration between infection and report parameters.

In Vietnam, it is possible to make rough estimates of the date an outbreak is removed from the day of report and other information such as official control policies and guidelines [30] (in the baseline model this is assumed to occur the day following the report of an outbreak). If the infection time of a commune was observed it would also be possible to make an estimate of the time a commune becomes infectious from previous experimental data [31]. However, the time between the infection of a commune and the report of an outbreak is likely to vary widely between each outbreak and depend upon a multitude of factors including the type of farm involved, the size of the flock, the species of bird involved, the background mortality previously experienced, the willingness to report disease and the level of infection in the vicinity of the infected premises. To overcome this problem a data augmentation approach was used, with unobserved infection times treated as nuisance parameters [13],[14]. Using Bayes' theorem, the joint posterior distribution of the model parameters and augmented infection times is:
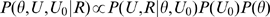
where 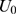 is the index infected commune, with 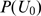
a flat prior. This was then sampled using a two-step MCMC algorithm, initiated by randomly drawing putative infection times for each outbreak from a uniform proposal in the interval between 1 and 30 days prior to date the outbreak was reported. The algorithm then consists of a standard random-walk Metropolis Hastings sampler to update the infectivity and time to report parameters, assuming flat prior distributions, and an independence sampler of individual infection times where the putative infection times of outbreak are repeatedly proposed and updated with probability
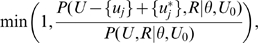
where 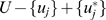 is the set of infection times with the existing imputed infection time of the selected commune 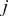 replaced with the proposed time. Thus the probability of accepting a proposed infection time takes into account both the estimated infection-to-report distribution and the location, removal times and imputed infection times of all other outbreaks (for further details of this algorithm see *Text S1*).

#### Simulation study

To assess the ability of the fitting procedure to accurately estimate transmission parameters, given the validity of all the modelling assumptions, outbreaks of HPAI were simulated over the spatial distribution of communes in Vietnam. This was done by seeding an outbreak within a commune in the high-risk Red River Delta region with an infectious period drawn from a predefined gamma distribution. Secondary infections were then chosen according to an infection probability as described in the model, calculated using a preselected set of parameter values and commune level poultry populations estimated from the 2005 poultry population data. Infectious periods for these communes were drawn from the same gamma distribution as before. This procedure was repeated at each time-step, representing a day of the outbreak wave, and infected communes were removed from the dataset at the end of their infectious periods. The simulation was continued until no infectious communes remained.

**Figure 5:**
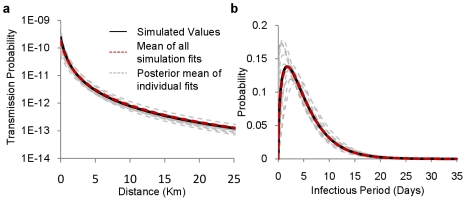
Simulation study results. Parameters used during simulation of outbreaks waves (black line), estimates from the posterior mean of the model parameters estimated from fitting the model to each simulated wave (dashed grey lines) and the overall mean of each of these estimates (dashed red line) for a, the commune-level infectious period and b, infectiousness over space as described by the product of *β* and the spatial kernel.

50 datasets were generated using the transmission kernel and infectious period shown in Fig. S3 and the 13 simulations where the size of the outbreak exceeded 20 communes were selected. Once simulated datasets had been obtained the fitting procedure was applied, using only the spatial location and date of report of each outbreak (entered as the day before the outbreak was removed). The ability of the fitting procedure to recapture the set of parameter values used to simulate the outbreak wave was then assessed (results shown in Fig. 5).

#### Calculating Risk maps

The local reproductive number of a commune estimated from an outbreak wave is defined as the number of secondary infections which could have been expected to occur had the first outbreak of the wave occurred in that particular commune, when all other communes remain susceptible As a result these estimates incorporate the effects of existing control measures. It can be obtained analytically [32] using the expression:
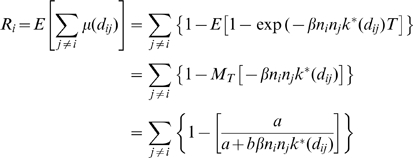
here 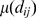 is the probability that transmission occurs between an infectious and susceptible commune 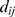 apart at some time during the infectious period, which is assumed to take the form of a gamma distribution, 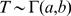, with 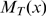 the moment generation function of this distribution. Similarly we calculate the expected number of infections which would occur on the first day of an outbreak, 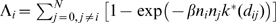.

### Reconstructing the epidemic process

By drawing samples of infection times from the MCMC output it is possible to calculate the marginal probability that any given outbreak is infected by any other outbreak: 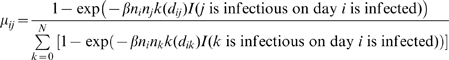
It is then possible to calculate both the expected number of infections arising from each individual outbreak commune and the expected distance over which infection was transmitted (see *Text S1*). Moreover, in order to assess the effects of earlier detection, realisations of the potential epidemic tree (i.e. the unambiguous chain of transmission where each outbreak is assigned a source infector) can be repeatedly sampled for each set of infection times by randomly selecting a source outbreak for each outbreak according to these probabilities. From this, secondary infections which arose between the “real-life” removal time of an outbreak and a putative earlier removal time, occurring as a result of improved surveillance, were identified, according to the assumed impact of this surveillance. Then, by pruning clades of outbreaks attributed to these secondary infections [16], the epidemic tree which would have occurred as a result of the earlier removal time is obtained (assuming pruned outbreaks would not have been infected from alternative sources at a latter stage of the wave). This form of analysis was used to assess the impact that the surveillance scenarios listed in Fig. 4 would have had upon the 2007 wave of outbreaks (see *Text S1*).

## Supporting Information

Text S1 Supplementary information includes further details of the computational methods used, in particular the MCMC model fitting and epidemic tree reconstruction algorithms, and a description of sensitivity analyses of the key results which were carried out.
(0.55 MB PDF)
