# Correction: A Bayesian Approach to Quantifying the Effects of Mass Poultry Vaccination upon the Spatial and Temporal Dynamics of H5N1 in Northern Vietnam

**DOI:** 10.1371/annotation/bdf0c45a-a656-4980-8bc8-7990255ed1ad

**Published:** 2010-03-11

**Authors:** Patrick G. T. Walker, Simon Cauchemez, Raphaëlle Métras, Do Huu Dung, Dirk Pfeiffer, Azra C. Ghani

The following information was missing from the Funding section: The authors are grateful to the UK Department for International Development (DFID) for funding this work through the HPAI risk reduction project (GCP/UK/804/INT).

