# Supplementary material for: A Bayesian Approach to Quantifying the Effects of Mass Poultry Vaccination upon the Spatial and Temporal Dynamics of H5N1 in Northern Vietnam
Source: PLoS Comput Biol. 2010 Feb 19;6(2):e1000683. doi: 10.1371/journal.pcbi.1000683 (PMC2824746; doi:10.1371/journal.pcbi.1000683)
Supplement: Text S1 — Supplementary information includes further details of the computational methods used, in particular the MCMC model fitting and epidemic tree reconstruction algorithms, and a description of sensitivity analyses of the key results which were carried out. (0.55 MB PDF) [file pcbi.1000683.s001.pdf]

A Bayesian approach to quantifying the effects of mass poultry vaccination upon the spatial and temporal dynamics of H5N1 in northern Vietnam

Text S1

Patrick G.T. Walker*, Simon Cauchemez, Raphaelle Metras , Do Huu Dung , Dirk Pfeiffer & Azra Ghani

***To whom correspondence should be addressed. E-mail: patrick.walker@imperial.ac.uk;**

Table of contents

1. Details of MCMC algorithm 3

2. Estimating the change in per-capita infectivity between waves......................................4

3. Comparing Risk Maps...5

4. Reconstructing the epidemic process...6

5. Evaluating the effects of more rapid detection and culling..6

6. Sensitivity analyses..7

i. 24 hour duration between report and removal...7

ii. Constant infectivity profile...9

iii. Impact of unobserved outbreaks..10

7. Bibliography...12

1. Details of MCMC algorithm

The posterior distribution was sampled by proceeding according to the following MCMC algorithm:

(1). Starting values of the kernel and time-to-report are assumed to follow a gamma distribution. The duration between infection and report for each outbreak are repeatedly drawn from a uniform proposal distribution of between 1 and 30 days until a possible augmented starting set of infection times (i.e. every outbreak except for the first to occur has a potential source of infection) is obtained.

(2). Each kernel and time to report parameter are updated using a standard, random-walk single-component Metropolis-Hastings algorithm where proposed updates are drawn on a log-scale from a normal distribution centred on the existing parameter value with a variance calibrated, by assessing the ratio of proposed moves which are accepted, so as to achieve good MCMC mixing (ideally as close to the 'golden' acceptance ratio of 23%[1] but a ratio of 10-50% was ajudged to represent adequate mixing).

(3). The duration between infection and report, and thus the infection time itself, of individual outbreaks are updated using a Metropolis-Hastings independence sampler[2] with the same proposal distribution as that used in step (1) to obtain a starting set. Using U now to denote the set of current infection times, the current infection time u_j of a randomly selected commune j is replaced with the proposed infection time u_j^* with probability

$$\frac{P\left(U-\{u_j\}+\{u_j^*\},R\middle|\theta,U_0\right)}{P(U,R|\theta,U_0)}\wedge 1$$

where $U-\{u_j\}+\{u_j^*\}$ denotes the set of infection times with the proposed replacing the current infection time. As the likelihood is conditional on the existence a unique initial infection, any move resulting in more than one initial outbreak was allocated zero likelihood and rejected. In order to achieve faster convergence it is desirable to update more than one infection time during this step (updating 10% of infection times appears to be a reasonably good rule of thumb). Evaluating the entire likelihood for each of these proposed time results in the algorithm becoming computationally intensive. In view of this the sampler was designed to calculate the acceptance probability by comparing the existing and proposed likelihood in the interval $\left[\min\left(u_j,u_j^*\right),\max\left(u_j,u_j^*\right)\right]$, the only section of the likelihood affected by the proposed move.

Steps (2) and (3) are repeated for a 'burn in' period of 20000 moves, determined by examining parameter traces from different starting parameter values, at which point the MCMC is deemed to have converged to its stationary distribution and subsequent moves are treated as samples of the

posterior distribution of each of the parameters. The chain is then run until its stopping time, determined by examining Monte Carlo standard errors and estimates of the posterior distribution of the parameters using different starting values, 250000 moves later. Resulting parameter estimates from two chains per wave, each run for the required 270000 moves of the MCMC algorithms from different starting values are presented in Table S1 and sample MCMC output in Fig. S1.

Fig. S1| Sample MCMC output. Plots show a, log likelihood and b, power kernel parameter α traces of the 2007 outbreak data under the baseline model assumption for two sets of initial parameter values: $\alpha = 1$, $\gamma = 1$, $\beta = 10^{-7}$, $R - U \sim \Gamma(3,3)$ (blue lines) and $\alpha = 10$, $\gamma = 100$, $\beta = 10^{-10}$, $R - U \sim \Gamma(6,1)$.

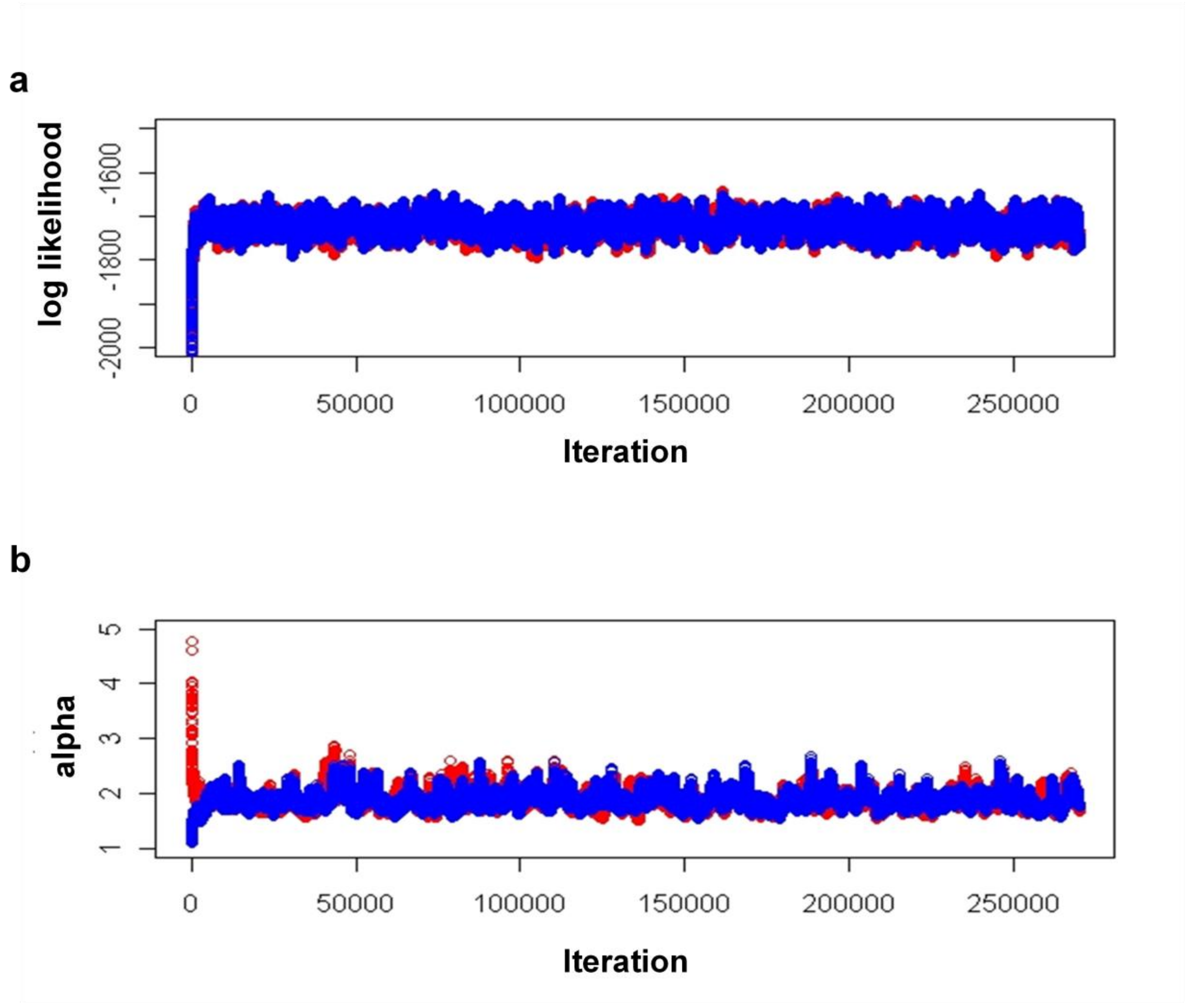

2. Estimating the change in infectivity between waves

Estimating the proportional change in infectivity between outbreaks is an easily interpretable measure of the incremental effect of control policies. This is particularly the case when estimating the overall protective effect of a vaccination campaign. One method for estimating this statistic from the fitting

procedure is to fit the model to two waves simultaneously, allowing only the kernel parameter β and the infection time parameters to vary between the waves.

3. Comparing Risk Maps

Where risk maps are being used to estimate the effects of control policies it is necessary to standardise the risk maps by using the same denominator poultry population for each set of posterior parameter distribution. Fig. S2 shows the results of comparing the posterior estimates of the model parameters obtained from 2004/5 and 2007 wave of outbreaks in this way, standardising to the 2007 estimates of poultry densities.

Fig. S2| Comparing local reproductive numbers before and after vaccination. Map highlighting communes where the mean posterior estimate of R exceeded 1 or 2 either only 2004/5 or only in 2007, with poultry populations standardised to those caluclated for 2007. Map shows an 11% (191 communes) reduction in number of communes with R>1 and a 14.1% (79 communes) reduction in those with R>2.

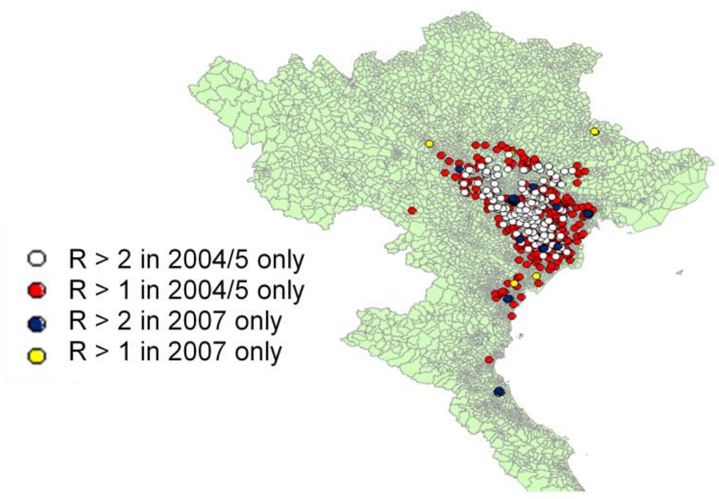

4. Reconstructing the epidemic process.

When the times at which a commune is infected and remains infectious are observed the reproductive number and the expected distance over which infection was transmitted can by estimated by reconstructing the epidemic tree[3]. The probability that commune i is infected by j is then

$$\mu_{ij} = \frac{1 - \exp\left(-\beta n_i n_j k(d_{ij})\right) I(j \text{ is infectious on day } i \text{ is infected})}{\sum_{k=0}^{N}[1 - \exp(-\beta n_i n_k k(d_{ik})) I(k \text{ is infectious on day } i \text{ is infected})]}$$

However, when the times of infection are not available a sufficiently large number of samples of μ_{ij} have to be drawn at regular intervals during the MCMC. The probability that i is infects j is then estimated by:

$$\mu_{ij}^{*} = \overline{\mu_{ij}}$$

where $\overline{\mu_{ij}}$ is the posterior mean of μ_{ij} estimated by calculating the mean of the obtained samples.

From this the expected distance D_i of an infected commune i from the commune which infected it can then be defined as

$$D_i = \sum_{j \in N} \mu_{ij}^{*} \, d_{ij}$$

Furthermore, the effective reproductive number of an infected commune i can be calculated by summing the infection probabilities between the commune and all potential offspring communes:

$$R_i = \sum_{j \in N} \mu_{ji}^{*}$$

This provides an indication of how the reproductive number changes as the outbreak wave progresses due to changes in the level of susceptibility within the communes around outbreaks and the application of control measures. If the reproductive number remains below unity for a sufficiently long period of time the epidemic has a high probability of dying out.

5. Evaluating the effects of more rapid detection and culling

Once the epidemic process has been reconstructed, the effect of detecting and removing an outbreak at an earlier time-point can be assessed. First a sample epidemic tree is drawn by assigning a source of infection for each outbreak probabilistically from the list of communes which were infectious on the day the outbreak commune was first infected. From this, secondary infections which arose between the “real-life” removal time of an outbreak and a putative earlier removal time, occurring as a result of improved surveillance, are identified, according to the assumed impact of this surveillance. Then, by pruning clades of outbreaks attributed to these secondary infections, the epidemic tree which would

have occurred as a result of the earlier removal time is obtained (assuming pruned outbreaks would not have been infected from alternative sources at a latter stage of the wave). Sampling epidemic trees repeatedly (100000 draws in our analysis) provides an estimate of the effects of the earlier detection for a given set of parameters and infection times and, in turn, repeating this for the sets of infection times and model parameters at regular intervals during the MCMC provides an estimate of the posterior distribution of the impact of this earlier detection.

We used this to estimate the size and duration of the wave which would have occurred in 2007 had the rate of detection been maintained to that estimated for either of the earlier waves in our analysis. To do this we calculated the percentile of the time to report distribution represented by the time between infection and report $r-u$, in each outbreak. The day the outbreak would have been detected during a previous wave, r^* was then calculated so that

$$P(T < r-u) = P(T^* < r^* - u)$$

where T and T^* are the time to report distributions of the 2007 and the earlier wave respectively, with the latter drawn randomly from the posterior distribution obtained from fitting the model to the outbreak data of that wave. The epidemic tree is then pruned according to this new set of removal times. This is then repeated 100000 times at regular intervals during the MCMC, randomly sampling a different T^* each time.

6. Sensitivity analyses

Here we assess the robustness of our main qualitative findings to the key assumption made during the model formulation:

i. 24 hour duration between report and removal

Thus far our analysis is based upon the assumption that outbreaks in all three of the waves are removed according to the guidelines distributed to veterinary paraprofessionals in the field[4] and that a combination of movement restrictions, quarantine measures and the immediate culling of flocks within which infection has been detected combine to ensure the outbreak is removed from the wave within 24 hours of the outbreak being reported. In reality, it is likely that it takes a longer time to achieve effective control in some, or even all cases. We assess the sensitivity of our results to this uncertainty by repeating the analysis with different report to cull durations. As expected, we found that as the assumed report to cull duration increased our estimates of the infection to report distribution decreased. However, as Fig. S4 illustrates, if the duration between report and removal remains constant across all three waves, our estimates still suggest that outbreaks were not detected as rapidly during the 2007 wave.

Whilst the estimate of the reduction in per-capita infectivity following vaccination (obtained by following the methodology detailed in section 5) is incrementally muted as the duration between reporting and removal is increased across the three waves (arising from the disproportionate increase in the estimated infectious period of the two earlier waves relative to that in 2007 (Fig. S4)), these estimates are still consistent with a reduction in infectivity and remain statistically significant until the report-to-removal delay reaches approximately ten days (Table S2).

Another plausible scenario we tested was that, as a possible consequence of it being a more complicated intervention to implement or the time needed for vaccine-induced immunity to be acquired[5], outbreaks in 2007 took a longer time to be effectively controlled by ring vaccination in comparison to the mass culling campaigns during the 04/05 and 2005 waves. However, we found that even if outbreaks took over a week longer to control in 2007, the estimated mean detection time remained greater than during the previous two waves (Fig. S4), whereas the reduction in infectivity became more pronounced (Table S2).

Fig. S3| Sensitivity analysis of the time between the report of an outbreak within a commune and the removal time of the outbreak. Estimated probability distribution of the duration between the beginning of an outbreak and the date it is reported (plotted values are the posterior mean parameter estimates) for the 2004/5 wave (red lines), 2005 wave (blue lines) and 2007 wave (green lines) under the assumption that communes remain infectious for at a constant level for 1 day (solid lines), 3 days (dashed lines) and 8 days (dotted lines).

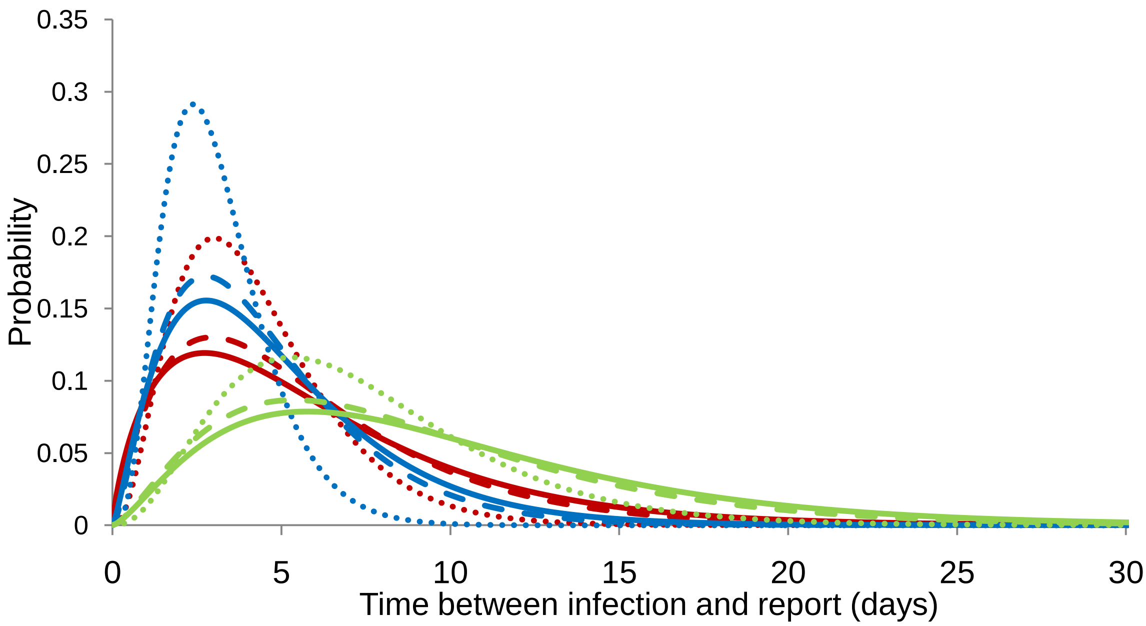

Table S1| Sensitivity analysis of the assumed duration between the report and the removal of an outbreak.

Time between report and removal during 04/05 wave	Time between report and removal during 2007 wave	Relative infectivity (95% C.I.s)
1 day	1 day	0.55 (0.37-0.8)
1 day	6 days	0.41 (0.28-0.58)
3 days	3 days	0.65 (0.45-0.91)
3 days	6 days	0.58 (0.38-7.5)
6 days	6 days	0.7 (0.53-0.96)
11 days	11 days	0.82 (0.63-1.01)

ii. Constant infectivity profile

As an alternative to the assumption that the communes remain infectious at a constant intensity throughout the duration of the infectious period, we explored a second scenario where we assumed infectiousness increased monotonically until the outbreak was detected, increasing rapidly during the early stages of infection but saturating quickly. For this we used the cumulative distribution function of an exponential distribution, with parameter δ calibrated so as to level off by the 10th day of the outbreak:

$$\beta(t-u) \propto 1 - \exp\left(-\frac{(t-u)}{\delta}\right).$$

Following the reporting of an outbreak the infectivity of the commune was then assumed to decay exponentially for the following week, with a 24 hour 'half-life'.

Fitting this infectivity curve to the outbreak data we once again found that the model estimated that, for the average duration of infection of the earlier waves, infectivity was lower following vaccination but that this was offset by a longer infectious period (Fig. S5).

Fig. S4| Modelling non-constant infectivity throughout the infectious period. Estimates of infectivity for the 2004/5 (red line), 2005 (blue line) and 2007 (green line) waves. Values shown correspond to an outbreak with wave specific mean infectious period and show the per-bird instantaneous probability of transmission to a susceptible commune 25km away (corresponding to the estimated median transmission distance calculated for the 2007 wave).

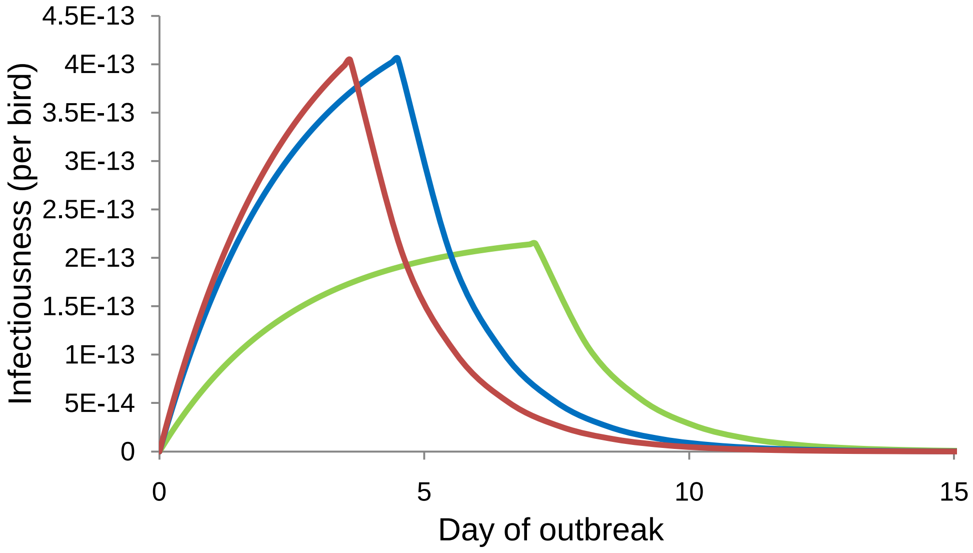

iii. Impact of unobserved outbreaks

Applying the fitting procedure to a wave where some outbreaks remain unobserved and unreported throughout the duration of infection would result in connecting outbreaks which are further apart in both time and space. As a result, especially at a high level of unreported outbreaks, the fitting procedure is likely to overestimate the commune-level infectious period. As this factor is of particular concern following a vaccination campaign where ‘silent spread’ may be an issue[6,7], we assessed whether it could explain the observed differences in the estimates of the infectious period and infectivity following vaccine.

We simulated an outbreak wave and then randomly selected unobserved outbreaks. Having selected a set percentage of unobserved outbreaks, we fitted the model to the remaining infections. We found that, as expected, the estimate of infectious period increases as the proportion of detected outbreaks decreases. However, as the wave is increasingly ‘thinned out’ in this way, the fitting procedure increasingly estimates transmission over longer distances. This produces a kernel which underestimates short range and overestimates long range transmissibility (Fig. S5). These results suggest that it is unlikely that an increased proportion of unobserved outbreaks during the 2007 wave

is the explanation for the reduction in the estimate of inter-commune transmissibility between waves whilst the notion of an increase in the level unobserved outbreaks does of course support the conclusion that there has been a decrease in overall detection capacity. It should be noted, however, that this assumes that the ability to detect outbreaks is uniform throughout Vietnam and throughout the wave of outbreaks and also that the infectious period of an outbreak would not affect, or be affected by, whether or not the outbreak was reported. In reality it is highly probable that this will not be the case. For example, short-lived outbreaks may be less likely to be detected and unreported outbreaks may remain infectious for a longer length of time than would have been the case had it been detected.

Fig. S5| Simulation study of the effect upon model estimates of underreporting. Figure showing a, how the probability of transmission scales by distance according to the spatial kernel and b, the commune level infectious period. Plotted values are those used to generate the simulated outbreak (dashed grey lines), those calculated using the posterior mean model parameter estimates obtained from the complete simulated dataset (solid black lines), the overall mean posterior parameter estimates from fitting the model to five datasets where 20% of outbreak are randomly not observed and therefore treated as susceptible throughout the wave (dashed black lines) and five datasets where 50% of outbreaks are randomly not observed.

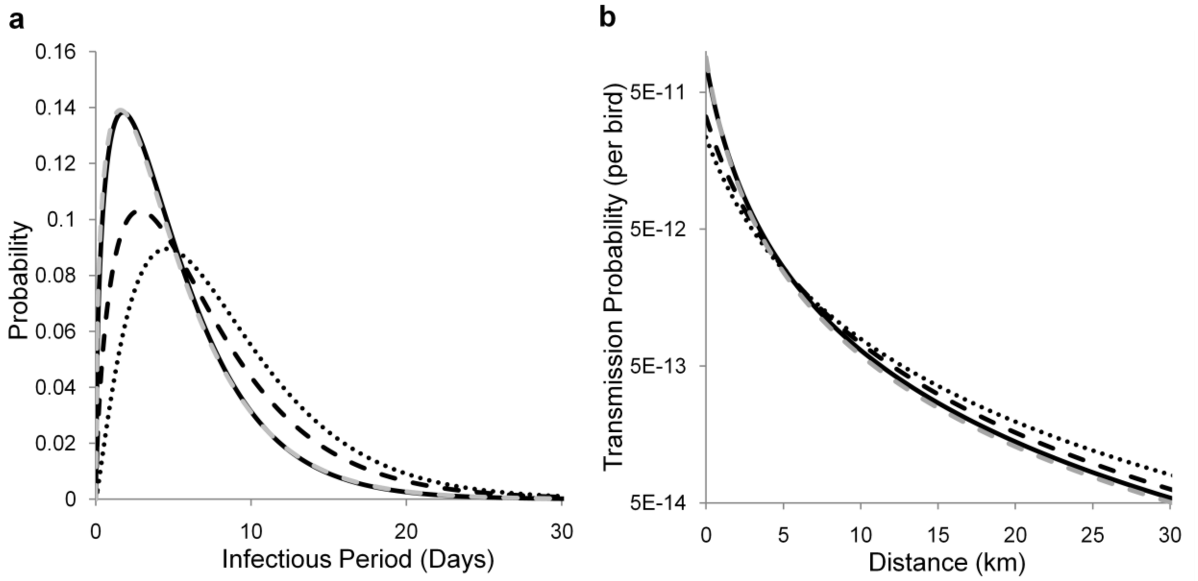

7. Bibliography

1. Roberts GO, Gelman A, Gilks WR (1997) Weak convergence and optimal scaling of random walk Metropolis Hastings algorithms. . Ann Appl Probab 7: 110-120.
2. Tierney L (1994) Markov Chains for Exploring Posterior Distributiona. The Annals of Statistics 22: 1701-1728.
3. Haydon DT, Chase-Topping M, Shaw DJ, Matthews L, Friar JK, et al. (2003) The construction and analysis of epidemic trees with reference to the 2001 UK foot-and-mouth outbreak. Proc Biol Sci 270: 121-127.
4. FAO (2006) Prevention and Control of Avian Flu in small scale poultry:A guide for veterinary paraprofessionnals in Vietnam. Rome: FAO.
5. Peyre M, Fusheng G, Desvaux S, Roger F (2009) Avian influenza vaccines: a practical review in relation to their application in the field with a focus on the Asian experience. Epidemiology and Infection 137: 1-21.
6. Webster RG, Peiris M, Chen HL, Guan Y (2006) H5N1 outbreaks and enzootic influenza. Emerging Infectious Diseases 12: 3-8.
7. Savill NJ, St Rose SG, Keeling MJ, Woolhouse MEJ (2006) Silent spread of H5N1 in vaccinated poultry. Nature 442: 757-757.
